# Liponeurocytoma: Rare Neoplasm of the Central Nervous System

**DOI:** 10.7759/cureus.59221

**Published:** 2024-04-28

**Authors:** Zachary Sokol, Peter Parsells, Ravichandra Madineni

**Affiliations:** 1 Lewis Katz School of Medicine, Temple University, Philadelphia, USA; 2 Neurosurgery, Main Line Health, Bryn Mawr, USA; 3 Neurosurgery, Thomas Jefferson University, Philadelphia, USA

**Keywords:** atypical tumor, who grade ii, neurosurgery, posterior cranial fossa tumor, liponeurocytoma

## Abstract

In this case report, we characterize an instance of diagnosis, treatment, characteristics, and outcomes of a patient with a liponeurocytoma, a rare WHO grade II brain tumor first described in 1978. This tumor has been described with a wide array of radiographic, microscopic, and histologic features, and there remains no consensus regarding the role of radiation therapy. Most patients have favorable outcomes after surgical resection. Here we present the case of a 46-year-old female who underwent suboccipital craniectomy for resection of a cerebellar mass, which was diagnosed as liponeurocytoma on final pathology. The patient experienced resolution of symptoms and is neurologically intact two years after resection of the tumor.

## Introduction

Liponeurocytoma is a rare, benign, WHO grade II neoplasm of the brain. Previously, it was considered a WHO grade I tumor, until its recategorization in 2007 after recurrence was noted. The first case was described in 1978 by Bechtel et al. as a “Mixed mesenchymal and neuroectodermal tumor of the cerebellum” [1]. Less than 100 cases have been described in the literature to date [1,2]. Little is known regarding the radiological, immunohistological, and pathologic features of this tumor, as varying and diverse characteristics have been reported. It is for these reasons that the diagnosis is difficult. Before the first description in 1978, other similar tumors were earlier described as “unusual medulloblastomas”, and “neurolipocytomas”. On CT, this tumor generally appears hypotenuse or isodense to surrounding tissue. On MRI, T1 is more commonly hypointense, although this has been shown to vary. On T2, it exhibits hyperintensity, and T1 +C shows heterogeneous enhancement [3]. Histologically, liponeurocytoma is characterized by lipidized intracellular deposits with interspersed neoplastic elements. Liponeurocytoma usually has positive immunohistochemical staining with synaptophysin, neuron-specific enolase (NSE), hexaribonucleotide binding protein 3 (NeuN), and glial fibrillary acidic protein (GFAP). Most, but not all liponeurocytomas are negative for tumor protein p53 (TP53), and neurofilament [3-5].

This tumor generally occurs between 40 and 50 years of age, although it has been reported in patients aged 4-77. There is a slightly higher incidence in women than men. These tumors most commonly occur unilaterally in the cerebellar hemispheres, although they have been known to arise in the fourth ventricle [2,4,5]. Typical presenting symptoms are related to the common location of the tumor in the cerebellum, and include headache, vomiting, nausea, dizziness, ataxia, gait disturbance, and falls. Occasionally, the tumor may obstruct CSF flow, leading to a presentation similar to obstructive hydrocephalus [2].

Limited research exists on these tumors, and there is no consensus on treatment, although surgery is considered to be the therapy of choice, with prognosis being generally positive following proper treatment. The rate of recurrence in the literature with surgery alone is quoted as 44% and is further reduced to 8% with adjuvant radiotherapy, however, no randomized controlled trials have been performed. Proper diagnosis of liponeurocytoma is critical, as this tumor is commonly mistaken for medulloblastoma or oligodendroglioma, for which the treatment is a combination of radio and chemotherapies with possible spinal axis radiation, which have significant side effects [2,3].

In this case report, we describe a case of a cerebellar liponeurocytoma and the management of the patient while reviewing relevant literature on this rare finding.

## Case presentation

A 46-year-old female with a past medical history of ovarian cysts presented to the emergency department complaining of a posterior headache for three months, which had worsened over the last two days with associated lightheadedness. She was evaluated by a neurologist and was treated with occipital block, muscle relaxant, and gabapentin, without relief. Due to worsening headaches, she underwent a non-contrast CT of the head, which revealed a left cerebellar mass with surrounding vasogenic edema and fourth ventricular effacement without hydrocephalus. The patient’s neurological exam remained unremarkable throughout this time period.

**Figure 1:**
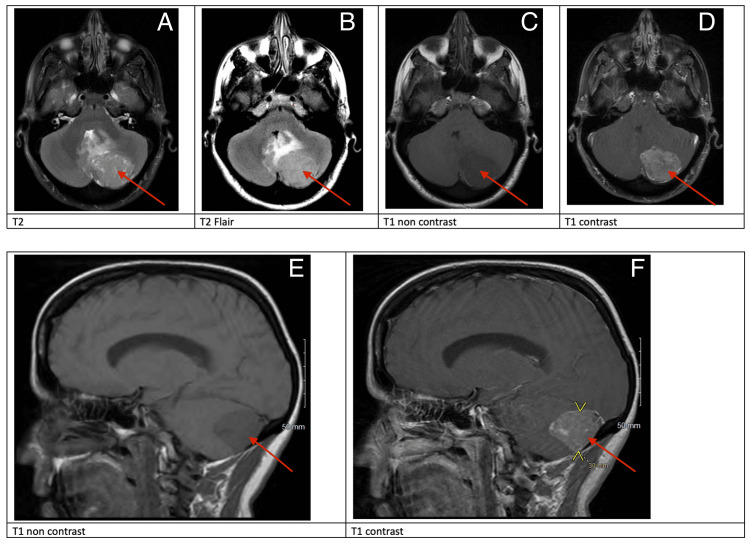
Preoperative MRI Red arrows: preoperative MRI demonstrating heterogeneous enhancement (C, D, E, F) and T2 (A) and T2 FLAIR (B) hyperintensity FLAIR: fluid-attenuated inversion recovery

MRI of the brain with and without gadolinium was performed, which demonstrated a left cerebellar extra-axial mass with vasogenic edema, mass effect on the fourth ventricle causing partial effacement, without hydrocephalus, and compression of the left transverse sinus without invasion (Figure 1). She was started on dexamethasone and acetaminophen and her symptoms improved. Metastatic workup with CT of the chest, abdomen, and pelvis with contrast showed no evidence of primary extracalvarial malignancy.

Left-sided suboccipital craniectomy with neuronavigation was performed with resection of the tumor. Navigation was used to delineate tumor boundaries and minimize risk to surrounding anatomy. After the dura was opened, the tumor was noted to be herniating out of the brain under pressure. The tumor appeared dark-colored and soft. Due to the consistency of the tumor, it was necessary to remove it piecemeal. Neuronavigation was used to confirm the boundaries of the tumor to ensure adequate dissection and removal. Postoperatively, the patient was neurologically stable with no new deficits.

**Figure 2:**
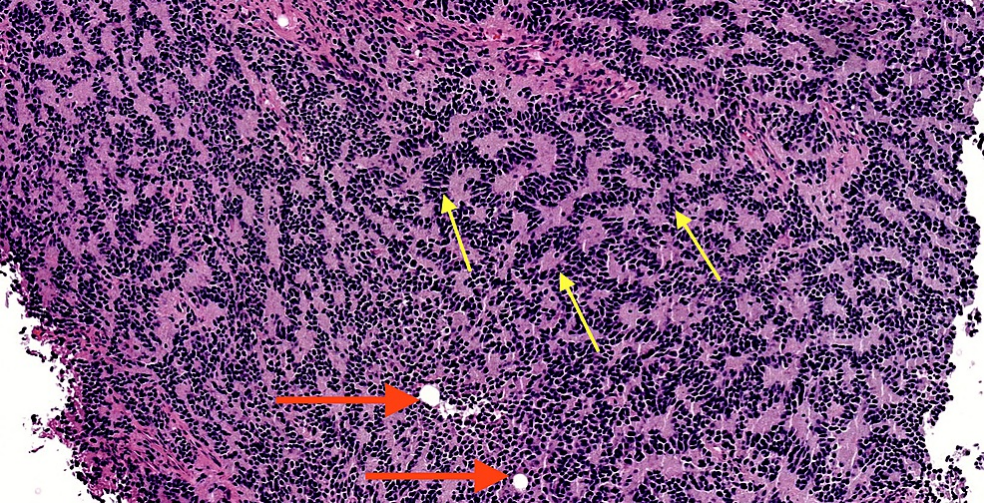
Histopathology images, H&E stain Yellow arrows: rosettes and palisading necrosis in a spongioblastoma-like pattern. Red arrows: Lipidized cells.

After gross total removal, the tumor was sent for interdepartmental pathology at a large academic center, which showed a heterogeneous neoplasm with prevalent neurocytic components. Of note were rosettes and palisading necrosis in a spongioblastoma-like pattern (Figure 2). A low level of mitotic activity was found. Immunostaining revealed positive focal GFAP, scattered S100, NeuN, INSM1, synaptophysin, and YAP1, and was weakly positive for TP53. Beta-catenin labeling revealed membranous findings. The neoplasm was negative for vimentin, CD31, CD34, GAB1, mutant IDH1 protein, and actin. Desmin was positive in large, focal cells. CD45 was positive in background lymphocytes. The Ki-67 index revealed patchy increases. Based on these findings, the neoplasm was categorized as heterogeneous, with the closest resemblance to cerebellar liponeurocytoma.

**Figure 3:**
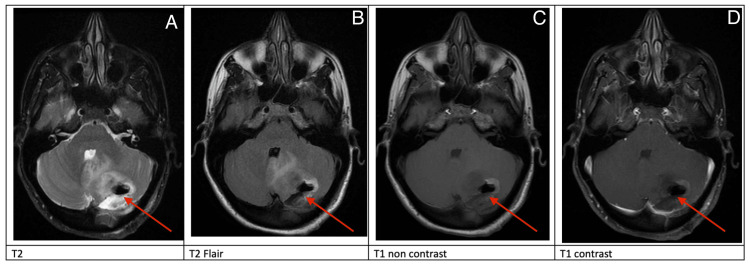
Postoperative day 1 MRI Postoperative day 1 MRI, red arrows (A-D): demonstrating gross total resection and expected postoperative changes

MRI of the brain with and without gadolinium was obtained on postoperative day #1, which showed a gross total resection with expected postoperative changes (Figure 3). She was discharged to home on postoperative day #2.

**Figure 4:**
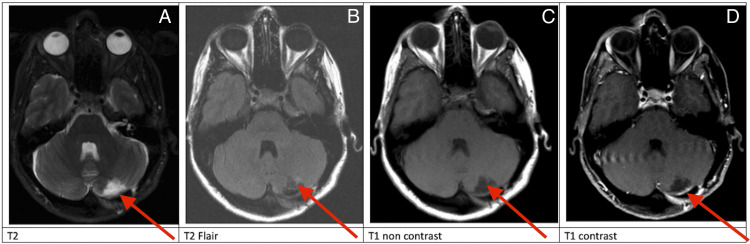
Three-month postop axial MRI Three-month postoperative MRI, red arrows (A-D) demonstrating no recurrent disease and stable resection cavity

It has been over two years since the time the patient underwent resection of her liponeurocytoma. The patient has returned to work and her preoperative recreational activities and remains symptom-free. She was followed for the first year with quarterly MRIs of the brain with and without gadolinium and will continue to be followed with an MRI of the brain every six months with and without gadolinium for the first five years (Figure 4). There have been no radiographic signs of recurrence at the time this case report was written.

## Discussion

Liponeurocytoma is a rare tumor with varied pathological and immunohistological findings [2-5]. Careful consideration of histological and immunohistological characteristics of the tumor must be considered during diagnosis, as improper diagnosis may lead to unnecessary radiation and chemotherapy [2,3].

Surgical resection is the first-line therapy, and there is no consensus on the role of adjuvant chemo-radiation therapies [2]. Radiation has been suggested in cases where the tumor cannot be completely removed, and some have opted for adjuvant radiotherapy as recurrence has been noted. Retrospective reviews have shown decreased recurrence after adjuvant radiotherapy, although no consensus exists [2,3]. In this case, the neoplasm was heterogeneous, with the greatest resemblance to liponeurocytoma. This is consistent with previously described liponeurocytomas in the literature, as there is diversity in the pathological and immunohistochemical features of this neoplasm with an average recurrence time of six years after resection [2,3,5]. The varied features of this neoplasm may account for the fact that multiple names existed for this tumor before its formal categorization in 1978. Characterization of this tumor remains difficult, as it may present with a diverse array of immunochemical markers, and the microscopic appearance may present with varying ratios of lipid, astrocytes, and neuroepithelial tissues [5].

If recurrence occurs, some authors have recommended repeat surgical resection, if possible, over treatment with radiation and/or chemotherapy [2]. Recurrence has not been reported in any patients who had complete resection and adjuvant radiotherapy, however, complete resection of the tumor has been shown to be the greatest factor in preventing recurrence in a single retrospective review [3]. The adjuvant radiation dose is reported as 54 Gy in most cases [3].

## Conclusions

Liponeurocytoma is a rare tumor most commonly occurring in the cerebellum, with various histological and pathological features. Less than 100 of these tumors have been reported in the literature. It is a benign tumor, and recurrence is uncommon; however, it has been reported. The role of radiation remains unclear. Further research is needed to further characterize this tumor in order to better understand and properly develop guidelines and recommendations for its management.

## References

[1] Chiaramonte C, Rabaste S, Jacquesson T, Meyronet D, Cotton F, Jouanneau E, Berhouma M (2018). Liponeurocytoma of the cerebellopontine angle. World Neurosurg.

[2] Gembruch O, Junker A, Mönninghoff C (2018). Liponeurocytoma: systematic review of a rare entity. World Neurosurg.

[3] Harrison W, Elsamadicy AA, McMahon JT, Chagoya G, Sobel RA, McLendon RE, Adamson C (2019). Glioneuronal tumor with features of ganglioglioma and neurocytoma arising in the fourth ventricle: a report of 2 unusual cases and a review of infratentorial gangliogliomas. J Neuropathol Exp Neurol.

[4] Owler BK, Makeham JM, Shingde M, Besser M (2005). Cerebellar liponeurocytoma. J Clin Neurosci.

[5] Xu L, Du J, Wang J, Fang J, Liu Z, He Y, Li G (2017). The clinicopathological features of liponeurocytoma. Brain Tumor Pathol.

